# Antimicrobial, Antibiofilm, and Antioxidant Properties of *Boletus edulis* and *Neoboletus luridiformis* Against Multidrug-Resistant ESKAPE Pathogens

**DOI:** 10.3389/fnut.2021.773346

**Published:** 2022-02-24

**Authors:** Juliana Garcia, Francisca Rodrigues, Flávia Castro, Alfredo Aires, Guilhermina Marques, Maria José Saavedra

**Affiliations:** ^1^CITAB – Centro de Investigação e Tecnologias Agroambientais e Biológicas, Universidade de Trás-os-Montes e Alto Douro, Vila Real, Portugal; ^2^Laboratório Colaborativo AquaValor Centro de Valorização e Transferência de Tecnologia da Água – Associação, Chaves, Portugal; ^3^REQUIMTE/LAQV - Polytechnic of Porto - School of Engineering, Porto, Portugal; ^4^CECAV, Veterinary and Animal Science Research Center, Universidade de Trás-os-Montes e Alto Douro, Vila Real, Portugal

**Keywords:** antibiotic resistance, wound infection, ESKAPE bacteria, wild mushroom, antibiofilm

## Abstract

Multidrug-resistant ESKAPE pathogens (*Enterococcus faecium, Staphylococcus aureus, Klebsiella pneumoniae, Acinetobacter baumannii, Pseudomonas aeruginosa*, and *Enterobacter species*) has become the most recurrent global cause of skin and soft-tissue infections, belonging to the WHO priority pathogens list. Successful therapy remains challenging and entails the assessment of novel and successful antibiotics. In this study, mushrooms are considered a valuable and unique source of natural antimicrobial compounds. Therefore, this study aimed to evaluate the antimicrobial and antibiofilm properties of *Boletus edulis* (*B. edulis*) and *Neoboletus luridiformis* (*N. luridiformis*) aqueous and methanolic extracts against ESKAPE isolates from clinical wound infections. Disk diffusion and microdilution methods were used to assess the antimicrobial activity. Phytochemical characterization was achieved by analysis of total phenols, orthodiphenols content, and antioxidant activity as well as by high-performance liquid chromatography-diode array detector (HPLC-DAD). Human foreskin fibroblasts-1 (HFF-1) cell viability was performed by the MTT assay. Aqueous and methanolic extracts of *B. edulis* and *N. luridiformis* showed antimicrobial and antibiofilm properties against multidrug-resistant bacteria, although with different efficacy rates. The results showed that there is a convincing relation between the content of phenolic compounds, antioxidant activity, and antimicrobial activity suggesting that the presence of phenolic compounds may explain the biological effects. HPLC analysis revealed high levels of protocatechuic acid, homogentisic acid, pyrogallol, gallic acid, p-catechin, and dihydroxybenzoic acid in the aqueous extract of *B. edulis*, explaining the highest antimicrobial and antibiofilm properties. Importantly, the mushrooms extracts were non-cytotoxic at all the tested concentrations. Overall, the tested mushrooms extracts are good candidates to further explore its use in the prevention of wound infection, particularly by multidrug-resistant pathogens.

## Introduction

Nowadays, the increase in multidrug-resistant (MDR) bacteria represents an important worldwide public health problem that inspires the search for new strategies to overcome this multifarious phenomenon (1). Several studies have showed that infections by MDR strains are one of the major causes of morbidity and mortality worldwide (2, 3). MDR bacteria are increasingly involved in infections associated with a variety of cutaneous wound types including burns, combat-related, surgical, and chronic wounds. Wounds are often characterized by a complex and potentially pathogenic microflora that presents a serious risk for both the wound infection and cross-contamination (4). The efficacy of antibiotics is influenced by several factors, namely, the wound microorganisms, their antibiotic-resistance profile, and the presence of bacteria-derived biofilm, which significantly enhances their tolerance to antibiotics (4). The most notorious MDR bacteria was identified as “ESKAPE,” which includes *Enterococcus faecium, Staphylococcus aureus, Klebsiella pneumoniae, Acinetobacter baumannii, Pseudomonas aeruginosa*, and *Enterobacter* species that are among the most prevalent bacteria in cutaneous infections (5). These ESKAPE pathogens have revived the attention on mushroom extracts as promising agents with antibacterial and antibiofilm activities. In fact, mushroom species contains several bioactive compounds, namely phenolic acids, terpenoids, flavonoids, tannins, alkaloids, and polysaccharides that could be used as novel and effective antibiotics (6, 7). On that basis, this study aims to chemically characterize different extracts of *Boletus edulis* (*B. edulis*) and *Neoboletus luridiformis* (*N. luridiformis*) as well as to evaluate its antioxidant properties. Moreover, the cytotoxicity, antimicrobial action, and ability to inhibit biofilm formation against clinical MDR isolates of ESKAPE pathogens were performed.

## Materials and Methods

### Chemicals and Drugs

Methanol of analytical grade purity was from Lab-Scan (Lisbon, Portugal). The culture media Brain Heart Infusion Agar (BHIA), Mueller Hinton Broth (MHB), Mueller Hinton Agar (MHA), and all the antibiotics were obtained from Oxoid (Hampshire, UK). The saline solution was prepared with NaCl from Merck (Darmstadt, Germany). The dye resazurin and crystal violet were purchased from Sigma-Aldrich (St Louis, Mosby, USA). All the organic solvents were HPLC grade. Ultrapure water from purified system (Isopad Isomantle, Gemini BV, Pr Beatrixlaan 301, 7312 DG Apeldoorn, Netherlands) was used. External standards caffeic acid, catechin, chlorogenic acid, ferulic acid, gallic acid, (-)-epicatechin, naringin, p-hydroxybenzoic acid, quercitrin, and rutin were purchased from Extrasynthese (Lyon Nord, Genay Cedex, France). Dulbecco's Modified Eagle Medium (DMEM), fetal bovine serum (FBS), non-essential amino acids, penicillin, streptomycin, and trypsin–EDTA were obtained from Invitrogen Corporation (Life Technologies, SA, Madrid, Spain).

### Mushrooms Material

*Boletus edulis* and *Neoboletus luridiformis* mushrooms were collected in a mixed stand at Sabugal, Guarda, located at the Center of Portugal. The morphological identification of the wild macrofungi was made till species according to macro- and microscopic characteristics (8). Representative voucher specimens were deposited at the mycological herbarium of Universidade de Trás-os-Montes e Alto Douro. After taxonomic identification, the mushrooms were immediately stored at −20°C, freeze dried (Dura Dry TM μP, −41°C and 500 mTorr), and grounded to a fine powder. The samples were kept in the dark in hermetically sealed plastic bags up to analysis.

#### Mushrooms Extracts

Mushrooms extracts were obtained by two different extraction methods according to our previous study (6): (1) Exhaustive aqueous extracts: 5 g of dried mushrooms material was added to 150 ml of distilled water. The mixture was agitated at room temperature (orbital shaker, 1 h, 150 rpm) and centrifuged. The supernatant was filtered (Whatman no. 4 filter paper) and 100 ml of distilled water was added to the pellet. The whole procedure was repeated 4 times. The total extracted was stored at −20°C before lyophilization to obtain the final extract; (2) Exhaustive methanolic extracts: 5 g of dried mushrooms material was added to 150 ml of 80% methanol solution (methanol/distilled water v/v). The mixture was agitated at room temperature (orbital shaker, 1 h, 150 rpm) and centrifuged. The supernatant was filtered and 100 ml of the previous solution was added to the pellet. The whole procedure was repeated four times. The total extracted volume was concentrated in a vacuum rotary evaporator at 38°C to remove methanol and stored at −20°C before lyophilization to obtain the final extract.

### Microorganisms and Culture Media

The microorganisms used were clinical isolates from patients hospitalized in various departments of Hospital Center of Trás-os-Montes and Alto Douro (CHTMAD)—these are located in the cities of Lamego, Peso da Régua, Chaves, and Vila Real, a Portuguese north province of Trás-os-Montes and Alto Douro. Ethical approval for this study was granted by the Ethics Committee of Hospital Vila Real (CHTMAD), according to a research collaboration protocol established in 2004. These strains belong to MJH and MJMC collections and are stored at −70°C in aliquots of BHI medium with 15% (v/v) glycerol in the Medical Microbiology Laboratory at Department of Veterinary Sciences — Antimicrobials, Biocides and Biofilms Unit at University of Trás-os-Montes and Alto Douro (UTAD). Several Gram-positive and Gram-negative bacteria isolated from wound exudates were used to screen the antimicrobial activity of the mushroom extracts. All the strains were identified by morphological and biochemical tests (morphological identification of colonies, Gram staining, conventional biochemical identification methods, and MicroScan WalkAway identification panels), followed by Kirby–Bauer antibiotic sensitivity assays, using different antibiotics. *Escherichia coli* (*E. coli*) (CETC 434) and *Staphylococcus aureus* (*S. aureus*) (CETC 976) strains were obtained from Spanish Type Culture Collection (CETC). Ethics approval for this study was granted by the Ethics Committee of Hospital Vila Real.

### Antimicrobial Activity

Briefly, bacterial suspension with the turbidity adjusted to 0.5 McFarland standard units, were spread with a sterile cotton swab onto Petri dishes (90 mm of diameter) containing 4 mm of Mueller-Hinton agar. Six-millimeter diameter sterile paper discs were dispensed on the seeded agar plates and imprinted with 12 μL of 1000 μg.mL^−1^ extracts solution (in Dimethyl Sulfoxide (DMSO) 10%). Incubation for 24 h at 37°C, followed by diameter measurement (mm) of the clear inhibitory zones around the discs imprinted with the extracts. In all experiments, a negative control (12 μL DMSO) and a positive control [standard commercial antibiotic gentamicin (10 μg)] were included.

The antimicrobial activity was classified according to the following scheme: noneffective (−): inhibition halo = 0; moderate efficacy (+): 0 < inhibition halo < antibiotic inhibition halo; good efficacy (++): antibiotic inhibition halo < inhibition halo < 2x inhibition halo; strong efficacy (+ + +): inhibition halo > 2x antibiotic inhibition halo (6). Minimum inhibitory concentration (MIC, lowest concentration of mushroom extract able to inhibit bacterial growth) was evaluated by a resazurin microdilution assay (6). Bacteria tested were picked from overnight cultures in Brain Heart Infusion. A small portion of bacteria was transferred into a bottle with 50 mL of Mueller Hinton Broth (MHB), capped and placed in an incubator overnight at 37°C. After 16 h of incubation, bacterial suspension was adjusted to an optical density of 0.5 measured at OD_500_ nm. The resazurin solution (3.4 mg.mL^−1^) was prepared in sterile distilled water. A 96-wells sterilized microplate was used and a volume of 100 μL of MHB was used in each well, together with 200 μL of extract solution, or positive control. From the first well (belonging to the first horizontal line) 100 μL was taken and added to the next well and then this step was repeated to each of the following wells in the vertical line, allowing a serial fold dilution of decreasing concentration (range of 1000 μg.mL^−1^ to 7.81 μg.mL^−1^). In addition, 20 μL of bacterial suspension and 20 μL of resazurin solution was added to each well. Microplates were incubated at 37°C for 18–24 h. All tests were performed in triplicate and MIC was then assessed visually by the color change of resazurin in each well (blue to pink in the presence of bacteria growth). For the determination of minimum bactericidal concentration (MBC, the lowest concentration of mushroom extract at which bacterial growth by at least 99.0%), the content from each well without changes in color was plated on Mueller-Hinton Agar and incubated at 37°C for 24 h. The lowest concentration that yielded no growth after this subculturing was taken as the MBC.

### Bacterial Adhesion/Biofilm Formation and Exposure to Extracts

The microtiter biofilm assay was used to assess the ability of the mushrooms extracts to control adhered cells of *S. aureus* CECT 976 and *E. coli* CECT 434 biofilms. Briefly, 96-well polystyrene microtiter plates were filled with 200 μl of bacterial suspension at OD_620_ nm of 0.04 ± 0.02 and incubated for 24 h (biofilm formation) at 37°C and 150 rpm. After the incubation period, the content of each well was aspirated and washed once with 200 μl of saline solution 0.85% (w/v). Then, 180 μl of fresh medium (MH broth) and 20 μl of extracts solution (to a final concentration at MIC, 5 × MIC, and 10 × MIC) were applied on biofilms. Concentrations ≥ MIC were used considering that biofilm-associated cells are usually 10–1,000-fold more resistant than in the planktonic state. Controls wells containing 10% (v/v) dimethyl sulfoxide (DMSO) were included. After 24 h of exposure at 37°C and 150 rpm, the effects of extracts were analyzed in terms of biomass and metabolic activity.

#### Biomass Quantification

After the treatment of biofilms with the different extracts, the content of each well of the microtiter plates was removed and the wells were washed with 250 μl of saline solution 0.85% (w/v) to remove non-adherent and weakly adherent bacteria. The remaining attached bacteria were fixed with 250 μl of 96% (v/v) ethanol and after 15 min, the microtiter plates were emptied. Then, 200 μl of 1% crystal violet (Merck, Portugal) were added to each well and allowed to stain for 5 min at room temperature. Afterward, the excess of crystal violet was gently withdrawn and 200 μl of 33% (v/v) glacial acetic acid (Fisher Scientific, UK) was added to solubilize the dye. Finally, the biomass was quantified by measuring the OD at 570 nm using a microplate reader. The results were presented as the biomass reduction (%) in relation to biofilms non-exposed to extracts (Equation 1).


(1) $$\begin{array}{r} \%\text{BR }=\;\left(\text{ODc }-\text{ ODw}\right)/\text{ODc}\times 100 \end{array}$$

where %BR is the percentage of biomass reduction, ODc is the OD_570_ nm value of control wells, and ODw is the OD_570_ nm value for the extract-treated wells.

#### Metabolic Activity Quantification

After the treatment of pre-established biofilms with mushroom extracts, the content of each well was removed and the wells were washed with 250 μl of saline solution 0.85% (w/v) to remove non-adherent and weakly adherent bacteria. For the staining procedure, 190 μl of fresh MH broth and 10 μl of resazurin solution at 400 μM were added to each well. Then, the microtiter plates were incubated for 20 min in the dark at room temperature. Metabolic activity was quantified by measuring the fluorescence at 570 and 590 nm, using a microtiter plate reader. The results were presented as metabolic inactivation (%) in relation to biofilms non-exposed to mushroom extracts (Equation 2).


(2) $$\begin{array}{r} \%\text{MI}=\left(\text{Fluoc }-\text{ Fluow}\right)/\text{Fluoc}\times 100 \end{array}$$

where %MI is the percentage of metabolic inactivation, Fluoc represents the fluorescence intensity of biofilms not exposed to extracts, and Fluow represents the fluorescence intensity value for biofilms exposed to extracts.

### Phytochemical Analysis

Phytochemical characterization of mushroom extracts was achieved by analysis of total phenols, orthodiphenols content, and antioxidant activity as well as by high-performance liquid chromatography-diode array detector (HPLC-DAD).

#### Determination of Total Phenolic Compounds

The total phenolic compounds in the extracts were determined by the Folin–Ciocalteu method as previous described (9). Briefly, 10 μl of mushroom extracts at a concentration of 1 mg ml^−1^ or gallic acid standards (0.01–1.0 mg ml^−1^ in methanol) were mixed with 185 μl of distilled water in a 96-well plate followed by the addition of 25 μl of Folin–Ciocalteu reagent. After an incubation period of 5 min, sodium carbonate (75 μl of 7% sodium carbonate) was added and further incubated for 2 h in the dark and at room temperature. The absorbance was then measured at 725 nm against a blank on the BioTek Powerwave XS2 Microplate Reader (BioTek Instruments Incorporation, Winooski, Vermont, USA) at 25°C. The phenolic content was expressed as mg gallic acid equivalents (GAE) per gram of extract dry weight (mg GAE/g DW).

#### Orthodiphenols Content

For the analysis of the orthodiphenols content, a colorimetric method, based on a complex reaction with sodium molybdate dehydrate, was applied (9). Briefly, extract aliquots (60 μl at a concentration of 1 mg ml^−1^) or gallic acid standards (0.01–1.0 mg ml^−1^ in methanol) were reacted for 25 min with 200 μl of a sodium molybdate dihydrate solution (5% prepared in ethanol/water, 1:1 v/v). The absorbance of the samples and standards was measured at 370 nm against a blank (ethanol/water 1:1, v/v) on a plate reader at 25°C. The results were expressed as mg of GAEs per gram of extract (mg GAE/g DW).

#### Determination of Antiradical and Antioxidant Capacities

To evaluate 2,2'-azinobis(3-ethylbenzothiazoline-6-sulfonic acid (ABTS) radical inhibition, 12 μl of sample or standard was placed in the microplate followed by 188 μl of ABTS + working solution. The plate was allowed to rest in the dark for 30 min at room temperature and the absorbance was read at 734 nm. The inhibition of ABTS + radicals was calculated using the following Equation (3).


(3) $$\begin{array}{r} \%\text{Inhibition }=\;\left(\text{Abs Blank }-\text{ Abs samples}\right)/\left(\text{Abs Blank}\right)\times 100 \end{array}$$

The antioxidant activity of the extracts was determined by interpolation of the calibration curve for Trolox, and the results were expressed in mmol Trolox per gram of DW [mmol Trolox/g DW; (9)].

To measure ferric reducing antioxidant power (FRAP), 20 μl of sample was placed in each well of the microplate followed by adding 180 μl of FRAP working solution. After incubating the reaction for 30 min at 37°C in the dark, absorbance was read at 593 nm. Trolox was used as standard and the results were expressed in mmol Trolox per gram of DW (mmol Trolox/g DW).

#### High-Performance Liquid Chromatography Analysis of Individual Polyphenol Compounds

The samples were submitted to HPLC-DAD in order to assess the profile and content of polyphenols, using the procedure next described, adapted from (10). A total of 500 μl of each extract obtained previously was injected in a system with an eluent composed by water with 0.1% of trifluoroacetic acid (TFA) (solvent A) and acetonitrile with 0.1% TFA (solvent B). The elution was performed at a flow rate of solvent of 1 ml min^−1^, with a gradient starting from 0% solvent B at 0 min, 0% solvent B at 5 min, 20% solvent B at 15 min, 50% solvent B at 30 min, 100% solvent B at 45 min, 100% solvent B at 50 min, 0% solvent B at 55 min, and 0% solvent B at 60 min. The injection volume was 10 μl of sample. Chromatograms were recorded at 254 and 280 nm for benzoic acids and flavan-3-ols, 320 nm for cinnamic acids, and 370 nm for flavonoids, with a C18 column (250, 9, 46 mm, 5 mm, ACE HPLC Columns, Advanced Chromatography Technologies Ltd, Abeerden, Scotland, UK). Individual polyphenols were identified using peak retention time, UV spectra, and UV maximum absorbance bands and trough comparison with external commercial standards (Extrasynthese, Cedex, France). External standards were freshly prepared in 70% methanol (methanol:water) at concentration of 1.0 mg ml^−1^ and running in HPLC-DAD simultaneously to the samples. The amount of each polyphenols was calculated using the internal standard (naringin) method. The results were expressed as mg 100 g^−1^ DW and calibration curves with standards were previously prepared and injected in HPLC in order to validate the method.

### Cytotoxicity Assay

Human foreskin fibroblasts-1 (HFF-1) was purchased from ATCC (ATCC Number: SCRC-1041; ATCC, Manassas, Virginia, USA). Cell line were grown in DMEM medium (Carlsbad, California, USA) fortified with L-glutamine, 10% inactivated fetal calf serum (FBS), antibiotic–antimitotic mixture (final concentration of 100 U/ml penicillin and 100 U/ml streptomycin) maintained in 5% CO_2_ incubator (Cell Culture^®^ CO_2_ Incubator, ESCO GB Ltd., UK). At 90–95% confluence, cells were trypsinized and plated in microtiter dishes. The viable cells were counted using trypan blue dye (Gibco) in hemocytometer. Cell viability was assessed using the (3-(4,5-dimethylthiazol-2-yl)-5-(3-carboxymethoxyphenyl)-2-(4 -sulfophenyl)-2H-tetrazolium (MTT purchased from Promega, Madison, Wisconsin, USA) conversion assay. Cells were cultured in 96-well microtiter plate at a density of 25 × 10^3^ cells per ml culture medium for 24 h. Then, cells were incubated with 1 μg.mL^−1^ to 10 mg.ml^−1^ of both the extracts and its corresponding formulation for 24 h at 37°C. After the removal of samples from the wells, cells were washed in phosphate-buffered saline, followed by addition of fresh medium. The microtiter plates were then incubated in a humidified atmosphere of 5% CO_2_ at 37°C for 24 h. To evaluate the number of viable cells, 100 μl of MTT solution was added into each well and incubated for 4 h at 37°C in the dark. Afterward, the medium was removed, the intracellular formazan crystals were solubilized, and extracted with 100 μl DMSO. After 15 min in continuous stirring at room temperature, the absorbance was measured at 490 nm with background subtraction at 630 nm in the Synergy HT Microplate Reader (BioTek Instruments Incorporation, Winooski, Vermont, USA; (11)).

### Statistical Analysis

Data are expressed as mean ± SD and were statistically analyzed by the one-way ANOVA, followed by the Holm-Sidak's multiple comparison test. Statistical analyses were performed using the GraphPad Prism software (San Diego, CA) for Windows (version 7) and differences were considered significant when *p* < 0.05 (95% significance).

## Results

### Antimicrobial Activity Evaluation of Extracts

Data on screening of antimicrobial activity of the B. edulis and N. luridiformis against Gram-positive and Gram-negative bacteria are shown in Tables 1, 2, respectively. Aqueous extracts from both the species of mushrooms represented high activity against most of the sensitive and resistant clinical isolates, while the methanolic extract showed a lesser activity against all the isolates. Concerning to aqueous extracts, the bacterial inhibition halos from Gram-positive varied between 8 and 25 mm and between 8 and 18 mm for *B. edulis* and *N. luridiformis*, respectively (Table 1). Noteworthy, aqueous extract also showed high bacterial inhibition halos in *P. aeruginosa*, with values varying between 13 and 21 mm and 7 and 17 mm for *B. edulis* and *N. luridiformis*, respectively (Table 2). Methanolic extracts from both the species showed lower bacterial inhibition halos values for all the clinical isolates. On the other hand, aqueous and methanolic extracts of both the species of mushrooms were not effective against *K. pneumoniae*.

**Table 1:** *In vitro* Gram-positive antimicrobial activity of positive control and aqueous and methanolic extracts of *B. edulis* and *N. luridiformis* (1,000 μg.mL^−1^), determined by the diameter of inhibition zones (mm).

			**Aqueous extrac**		**Methanolic extrac**		**Control**	
	**Bacterial isolates**	**Code**	***B. edulis***	***N. luridiformis***	***B. edulis***	***N. luridiformis***	**CN**	**DMSO**
Gram ^+^	MS *Staphylococcus aureus*	MJMC 018	9 ± 2.82 (+)	8 ± 0.71 (+)	7 ± 0.0 (+)	6 ± 0.0 (−)	S ^20 ± 1.0^	6 ± 0.0
	MR *Staphylococcus aureus*	MJMC 025	12 ± 2.12 (+)	10 ± 0.71 (+)	7 ± 0.0 (+)	6 ± 0.0 (−)	S ^17 ± 0.5^	
	MS *Staphylococcus aureus*	MJMC 026	12 ± 0.71 (+)	10 ± 0.71 (+)	6 ± 0.0 (−)	6 ± 0.0 (−)	S ^21 ± 0.0^	
	MS *Staphylococcus aureus*	MJMC 027	10 ± 1.41 (+)	10 ± 0.71 (+)	9 ± 0.0 (+)	6 ± 0.0 (−)	S ^17 ± 1.0^	
	MR *Staphylococcus aureus*	MJMC 102	18 ± 0.0 (+)	11 ± 0.71 (+)	8 ± 0.71 (+)	6 ± 0.0 (−)	S ^18 ± 2.0^	
	MS *Staphylococcus aureus*	MJMC 109	18 ± 1.41 (+)	13 ± 1.41 (+)	10 ± 2.12 (+)	8 ± 1.41 (+)	S ^20 ± 0.5^	
	MS *Staphylococcus aureus*	MJMC 110	10 ± 1.41 (+)	6 ± 0.0 (−)	9 ± 2.12 (+)	7 ± 2.12 (+)	S ^20 ± 0.5^	
	MR *Staphylococcus aureus*	MJMC 111	10 ± 0.70 (+)	8 ± 0.0 (+)	8 ± 0.71 (+)	6 ± 0.0 (−)	S ^16 ± 1.0^	
	MR *Staphylococcus aureus*	MJMC 507	10 ± 0.0 (+)	6 ± 0.0 (−)	6 ± 0.0 (−)	6 ± 0.0 (−)	S ^17 ± 2.0^	
	MS *Staphylococcus aureus*	MJMC 511	15 ± 0.71 (+)	11 ± 1.41 (+)	6 ± 0.0 (−)	6 ± 0.0 (−)	S ^20 ± 0.5^	
	MS *Staphylococcus aureus*	MJMC 534-B	18 ± 0.71 (+)	6 ± 0.0 (−)	6 ± 0.0 (−)	6 ± 0.0 (−)	S ^21 ± 0.5^	
	MR *Staphylococcus aureus*	MJMC 539	8 ± 1.41 (+)	6 ± 0.0 (−)	6 ± 0.0 (−)	6 ± 0.0 (−)	S ^19 ± 1.0^	
	MR *Staphylococcus aureus*	MJMC 545	9 ± 2.12 (+)	8 ± 0.71 (+)	6 ± 0.0 (−)	6 ± 0.0 (−)	S ^20 ± 1.0^	
	MR *Staphylococcus aureus*	MJMC 552	20 ± 1.41 (++)	9 ± 0.71 (+)	11 ± 0.71 (+)	9 ± 0.71 (+)	S ^18 ± 0.0^	
	MR *Staphylococcus aureus*	MJMC 565-A	13 ± 1.41 (+)	8 ± 0.71 (+)	6 ± 0.0 (−)	6 ± 0.0 (−)	S ^21 ± 2.0^	
	MR *Staphylococcus aureus*	MJMC 583	25 ± 2.12 (++)	18 ± 0.71 (++)	7 ± 0.0 (+)	6 ± 0.0 (−)	S ^17 ± 1.0^	
	MR *Staphylococcus aureus*	MJMC 605	8 ± 0.71 (+)	13 ± 1.41 (+)	6 ± 0.0 (−)	6 ± 0.0 (−)	S ^22 ± 1.0^	
	MS *Staphylococcus aureus*	MJMC 606	8 ± 0.71 (+)	6 ± 0.0 (−)	6 ± 0.0 (−)	6 ± 0.0 (−)	S ^18 ± 2.0^	
	MR *Staphylococcus aureus*	MJMC 615	9 ± 1.41 (+)	6 ± 0.0 (−)	6 ± 0.0 (−)	6 ± 0.0 (−)	S ^19 ± 1.0^	
	*Staphylococcus aureus*	CETC 976	9 ± 2.82 (+)	6 ± 0.0 (−)	6 ± 0.0 (−)	6 ± 0.0 (−)	S ^20 ± 1.0^	

*CN, Gentamicin; MR, methicillin-resistant; MS, methicillin-susceptible*.

**Table 2:** *In vitro* Gram-negative antimicrobial activity of positive control and aqueous and methanolic extracts of *B. edulis* and *N. luridiformis* (1,000 μg.mL^−1^), determined by the diameter of inhibition zones (mm).

			**Aqueous extrac**		**Methanolic extrac**		**Control**	
	**Bacterial isolates**	**Code**	***B. edulis***	***N. luridiformis***	***B. edulis***	***N. luridiformis***	**CN**	**DMSO**
Gram ^−^	*Klebsiella pneumoniae*	MJMC 537	6 ± 0.0 (−)	6 ± 0.0 (−)	6 ± 0.0 (−)	6 ± 0.0 (−)	S ^22 ± 0.5^	6 ± 0.0
	*Klebsiella pneumoniae*	MJMC 542	6 ± 0.0 (−)	6 ± 0.0 (−)	6 ± 0.0 (−)	6 ± 0.0 (−)	S ^21 ± 0.5^	
	*Klebsiella pneumoniae*	MJMC 543	6 ± 0.0 (−)	6 ± 0.0 (−)	6 ± 0.0 (−)	6 ± 0.0 (−)	S ^20 ± 0.0^	
	*Klebsiella pneumoniae*	MJMC 555	6 ± 0.0 (−)	6 ± 0.0 (−)	6 ± 0.0 (−)	6 ± 0.0 (−)	S ^23 ± 0.5^	
	*Klebsiella pneumoniae*	MJMC 562-B	6 ± 0.0 (−)	6 ± 0.0 (−)	6 ± 0.0 (−)	6 ± 0.0 (−)	S ^21 ± 0.5^	
	*Klebsiella pneumoniae*	MJMC 566	6 ± 0.0 (−)	6 ± 0.0 (−)	6 ± 0.0 (−)	6 ± 0.0 (−)	S ^21 ± 1.0^	
	*Klebsiella pneumoniae*	MJMC 569	6 ± 0.0 (−)	6 ± 0.0 (−)	6 ± 0.0 (−)	6 ± 0.0 (−)	S ^20 ± 0.5^	
	*Klebsiella pneumoniae*	MJMC 592	6 ± 0.0 (−)	6 ± 0.0 (−)	6 ± 0.0 (−)	6 ± 0.0 (−)	S ^22 ± 1.0^	
	*Klebsiella pneumoniae*	MJMC 597	6 ± 0.0 (−)	6 ± 0.0 (−)	6 ± 0.0 (−)	6 ± 0.0 (−)	S ^21 ± 0.0^	
	*Klebsiella pneumoniae*	MJMC 525	6 ± 0.0 (−)	6 ± 0.0 (−)	6 ± 0.0 (−)	6 ± 0.0 (−)	S ^22 ± 0.5^	
	*Acinetobacter. baumanni*	MJMC 561	6 ± 0.0 (−)	6 ± 0.0 (−)	6 ± 0.0 (−)	6 ± 0.0 (−)	S ^25 ± 0.5^	
	*Acinetobacter baumanni*	MJMC 526	6 ± 0.0 (−)	6 ± 0.0 (−)	6 ± 0.0 (−)	6 ± 0.0 (−)	S ^22 ± 1.0^	
	*Pseudomonas aeruginosa*	MJMC 540	21 ± 0.71 (+)	17 ± 0.71 (+)	13 ± 1.41 (+)	10 ± 1.41 (+)	S ^25 ± 0.2^	
	*Pseudomonas aeruginosa*	MJMC 553	18 ± 1.41 (+)	12 ± 0.71 (+)	14 ± 2.12 (+)	12 ± 0.70 (+)	S ^22 ± 0.0^	
	*Pseudomonas aeruginosa*	MJMC 559	20 ± 1.41 (++)	9 ± 0.71 (+)	6 ± 0.0 (−)	6 ± 0.0 (−)	S ^17 ± 0.5^	
	*Pseudomonas aeruginosa*	MJMC 568-A	13 ± 1.41 (+)	8 ± 0.71 (+)	6 ± 0.0 (−)	6 ± 0.0 (−)	S ^25 ± 1.0^	
	*Pseudomonas aeruginosa*	MJMC 598	22 ± 1.41 (+)	13 ± 1.41 (+)	13 ±0.70 (+)	9 ±0.70 (+)	S ^22 ± 1.0^	
	*Escherichia coli*	CETC 434	13 ± 1.41 (+)	7 ± 0.0 (+)	9 ±0.70 (+)	7 ±0.0 (+)	S ^18 ± 0.5^	

*CN, Gentamicin*.

The MIC and MBC of extracts against Gram-positive and Gram-negative clinical isolates are given in Tables 3, 4, respectively. The MIC values for Gram-positive bacteria ranged between 15.63 and 62.5 μg.ml^−1^ and 62.5 and 250 μg.ml^−1^ for aqueous *B. edulis* and *N. luridiformis*, respectively. The MIC values for *P. aeruginosa* varied from 7.81 to 62.5 μg.ml^−1^ and 31.25 to 125 μg.ml^−1^ for aqueous *B. edulis* and *N. luridiformis*, respectively. Methanolic extracts from both the species showed lower MICs values for all the clinical isolates. Similar to diffusion test, aqueous and methanolic extracts of both the species were not effective against *K. pneumoniae*.

**Table 3:** Minimum inhibitory concentration (μg.mL^−1^) and minimum bactericidal concentration for aqueous and methanolic extracts of *B. edulis* and *N. luridiformis*.

			**Aqueous extract**				**Methanolic extracts**			
			***B. edulis***		***N. luridiformis***		***B. edulis***		***N. luridiformis***		**CN**
	**Bacterial isolates**	**Code**	**MIC**	**MBC**	**MIC**	**MBC**	**MIC**	**MBC**	**MIC**	**MBC**	**MIC**
GRAM ^+^	*MS Staphylococcus aureus*	MJMC 018	31.25	500	125	>1,000	500	1,000	1,000	>1,000	62.5
	*MR Staphylococcus aureus*	MJMC 025	62.5	1,000	125	>1,000	500	1,000	1,000	>1,000	62.5
	*MS Staphylococcus aureus*	MJMC 026	31.25	500	250	>1,000	500	1,000	1,000	>1,000	15.63
	*MR Staphylococcus aureus*	MJMC 027	31.25	500	250	>1,000	250	>1,000	500	>1,000	15.63
	*MR Staphylococcus aureus*	MJMC 102	62.5	>1,000	125	>1,000	250	>1,000	500	>1,000	15.63
	*MS Staphylococcus aureus*	MJMC 109	31.25	500	62.5	500	250	1,000	500	1,000	15.63
	*MS Staphylococcus aureus*	MJMC 110	15.63	250	62.5	500	125	500	250	1,000	7.81
	*MR Staphylococcus aureus*	MJMC 111	15.63	250	62.5	500	125	500	500	1,000	7.81
	*MR Staphylococcus aureus*	MJMC 507	62.5	500	125	>1,000	250	1,000	250	>1,000	62.5
	*MS Staphylococcus aureus*	MJMC 511	31.25	500	125	>1,000	500	1,000	1,000	>1,000	7.81
	*MR Staphylococcus aureus*	MJMC 534B	15.63	500	250	>1,000	500	>1,000	1,000	>1,000	7.81
	*MR Staphylococcus aureus*	MJMC 539	125	500	250	500	500	1,000	1,000	1,000	62.5
	*MR Staphylococcus aureus*	MJMC 545	31.25	500	62.5	>1,000	250	>1,000	500	>1,000	62.5
	*MR Staphylococcus aureus*	MJMC 552	31.25	1,000	62.5	>1,000	250	>1,000	250	>1,000	15.63
	*MR Staphylococcus aureus*	MJMC 565A	15.63	250	125	500	125	500	250	1,000	15.63
	*MR Staphylococcus aureus*	MJMC 583	62.5	500	125	1,000	125	500	500	1,000	31.25
	*MR Staphylococcus aureus*	MJMC 605	62.5	>1,000	250	>1,000	250	500	500	500	62.5
	*MS Staphylococcus aureus*	MJMC 606	125	>1,000	250	>1,000	500	500	1,000	500	31.25
	*MR Staphylococcus aureus*	MJMC 615	125	500	250	1,000	500	>1,000	1,000	>1,000	62.5
	*Staphylococcus aureus*	CETC 976	62.5	500	125	1,000	500	>1,000	1,000	>1,000	15.63

*CN, Gentamicin; MR, methicillin-resistant; MS, methicillin-susceptible*.

**Table 4:** Minimum inhibitory concentration (μg.mL^−1^) and minimum bactericidal concentration for aqueous and methanolic extracts of *B. edulis* and *N. luridiformis*.

			**Aqueous extract**				**Methanolic extracts**				
			***B. edulis***		***N. luridiformis***		***B. edulis***		***N. luridiformis***		**CN**
	**Bacterial isolates**	**Code**	**MIC**	**MBC**	**MIC**	**MBC**	**MIC**	**MBC**	**MIC**	**MBC**	**MIC**
GRAM	*Klebsiella pneumoniae*	MJMC 537	NI	ND	NI	ND	NI	ND	NI	ND	7.81
	*Klebsiella pneumoniae*	MJMC 542	NI	ND	NI	ND	NI	ND	NI	ND	7.81
	*Klebsiella pneumoniae*	MJMC 543	NI	ND	NI	ND	NI	ND	NI	ND	62.5
	*Klebsiella pneumoniae*	MJMC 555	NI	ND	NI	ND	NI	ND	NI	ND	62.5
	*Klebsiella pneumoniae*	MJMC 562-B	NI	ND	NI	ND	NI	ND	NI	ND	31.25
	*Klebsiella pneumoniae*	MJMC 566	NI	ND	NI	ND	NI	ND	NI	ND	31.25
	*Klebsiella pneumoniae*	MJMC 569	NI	ND	NI	ND	NI	ND	NI	ND	31.25
	*Klebsiella pneumoniae*	MJMC 592	NI	ND	NI	ND	NI	ND	NI	ND	62.5
	*Klebsiella pneumoniae*	MJMC 597	NI	ND	NI	ND	NI	ND	NI	ND	62.5
	*Acinetobacter baumanni*	MJMC 525	NI	ND	NI	ND	NI	ND	NI	ND	15.63
	*Acinetobacter baumanni*	MJMC 561	NI	ND	NI	ND	NI	ND	NI	ND	15.63
	*Pseudomonas aeruginosa*	MJMC 526	15.63	250	125	500	250	500	500	1,000	62.5
	*Pseudomonas aeruginosa*	MJMC 540	7.81	125	31.25	500	125	500	500	>1,000	31.25
	*Pseudomonas aeruginosa*	MJMC 553	31.25	500	62.5	>1,000	125	1,000	250	1,000	62.5
	*Pseudomonas aeruginosa*	MJMC 559	31.25	500	62.5	1,000	125	>1,000	250	500	31.25
	*Pseudomonas aeruginosa*	MJMC 568-A	62.5	1,000	125	>1,000	250	>1,000	500	1,000	31.25
	*Pseudomonas aeruginosa*	MJMC 598	15.63	250	125	500	250	1,000	500	>1,000	15.63
	*Escherichia coli*	CETC 434	62.5	>1,000	125	>1,000	500	1,000	1,000	>1,000	15.63

*CN, Gentamicin; NI, Not inhibited; ND, Not determined*.

Concerning to MBC, the lowest concentration with bactericidal effect of aqueous and methanolic extracts of *B. edulis* against Methicillin-resistant Staphylococcus aureus (MRSA) were 250 and 500 μg.ml^−1^, respectively. For *P. aeruginosa*, the values were 125 μg.ml^−1^ for aqueous *B. edulis* extract and 500 μg.ml^−1^ for methanolic extract. With respect to *N. luridiformis*, the MBC of aqueous and methanolic extracts against MRSA was 500 μg.ml^−1^. Similar results were achieved for *P. aeruginosa* with *N. luridiformis* aqueous and methanolic extracts.

### Effect of Extracts on Biofilms

**Figure 1:**
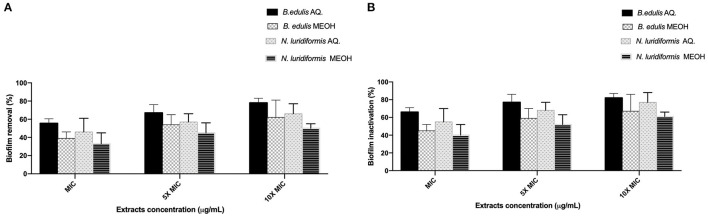
Effect of extracts at MIC, 5 × MIC, and 10 × MIC biofilms of *Staphylococcus aureus* CETC 976. **(A)** Percentage of biofilm removal, **(B)** Biofilm inactivation. Mean values ± SD for three independent experiments are illustrated.

**Figure 2:**
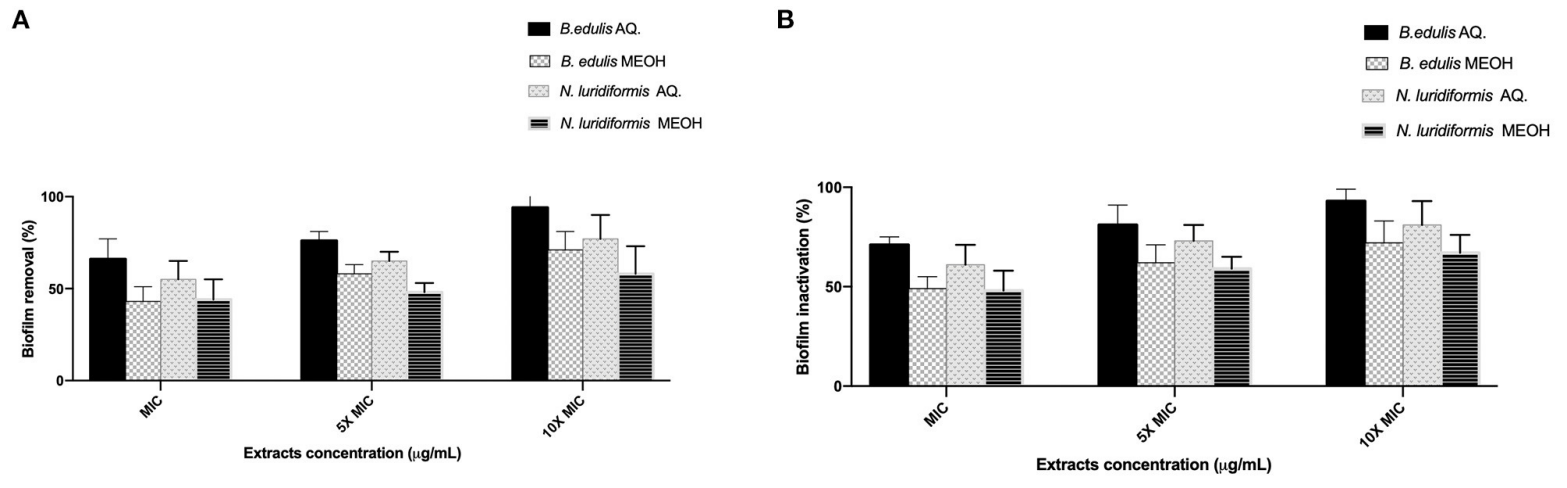
Effect of extracts at MIC, 5 × MIC, and 10 × MIC biofilms of *Escherichia coli* CETC 434. **(A)** Percentage of biofilm removal, **(B)** Biofilm inactivation. Mean values ± SD for three independent experiments are illustrated.

The efficacy of the different extracts at MIC, 5 × MIC, and 10 × MIC was tested against *S. aureus* CETC 976 and *E. coli* CETC 434 for 24 h and the biofilms were characterized in terms of biomass and metabolic activity (Figures 1, 2, respectively). The higher biomass removal was obtained for the aqueous extract of *B. edulis* in *S. aureus* (78.0 ± 5.02%) and in *E. coli* (94.0 ± 8.0%) at 10 × MIC (Figures 1A,B). Concerning the remaining analyzed extracts, the biofilm removal in both the bacterial species presented the following order: *N. luridiformis* aqueous > *B. edulis* methanolic > *N. luridiformis* methanolic for each concentration tested (Figures 1, 2). Concerning to the biofilm inactivation, the treatments with the different extracts caused the highest inactivation and presented the following order for both the biofilms (*S. aureus* and *E. coli*): *B. edulis* aqueous > *N. luridiformis* aqueous > *B. edulis* methanolic > *N. luridiformis* methanolic for each concentration tested (Figures 1, 2). The percentage of biofilm inactivation with the different extracts at 5 × MIC and 10 × MIC was higher than with MIC.

### Phytochemical Profile

#### Total Phenolic and Orthodiphenols Contents

**Figure 3:**
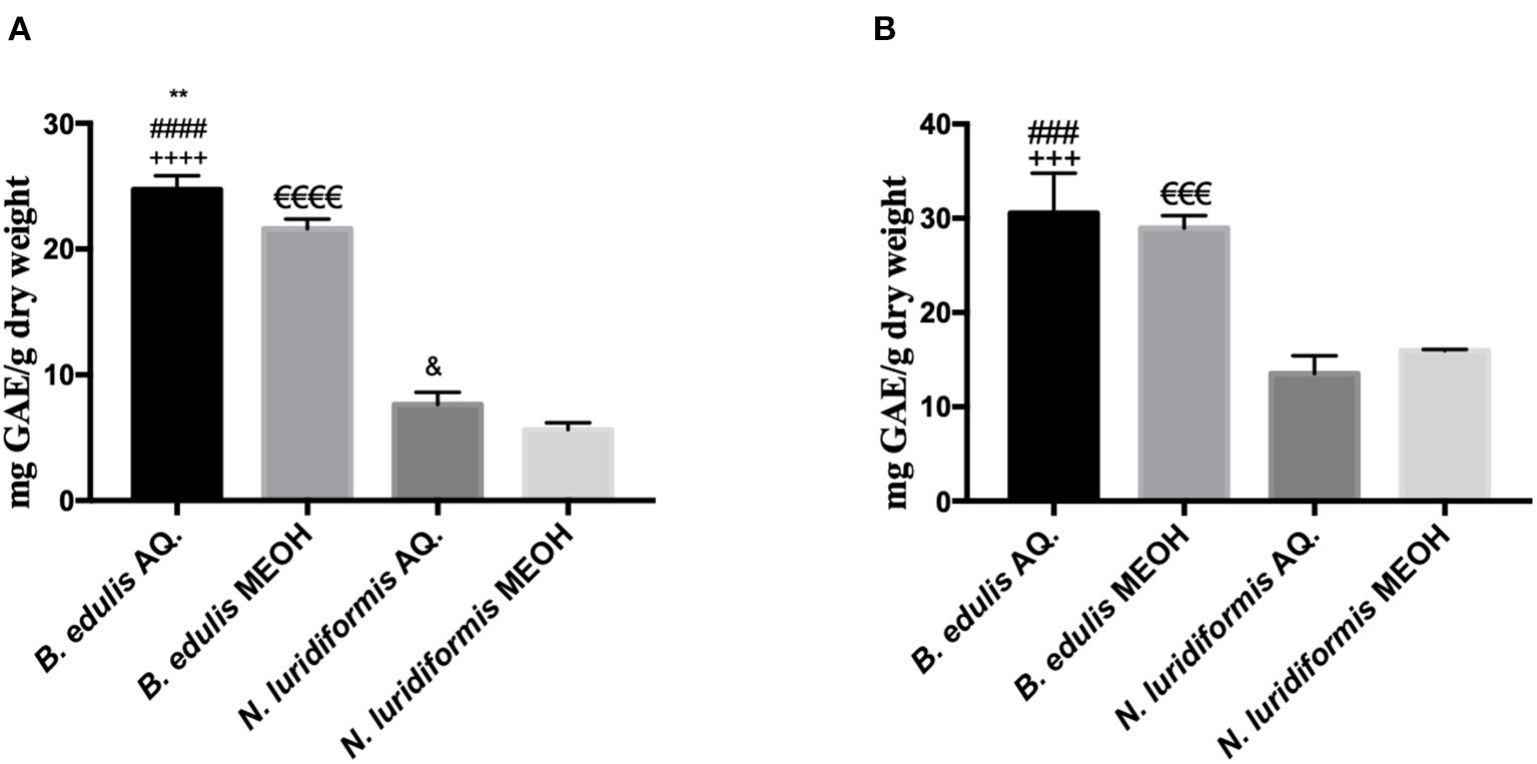
**(A)** Total phenolic content of aqueous (AQ.) and hydromethanolic (MEOH) extracts of *Boletus* edulis (*B. edulis*) and *Neoboletus luridiformis* (*N. luridiformis*) (**p < 0.0051 vs. *B. edulis* MEOH, ^####^*p* < 0.0001 vs. *N. luridiformis* AQ., ^++++^*p* < 0.0001 vs. *N. luridiformis* MEOH, ^e*eee*^*p* < 0.0001 vs. *N. luridiformis* MEOH, ^&^*p* < 0.0245 vs. *N. luridiformis* MEOH); **(B)** Orthodiphenols content of AQ. and MEOH extracts of *B. edulis* and *N. luridiformis* (^###^*p* < 0.0001 vs. *N. luridiformis* AQ., ^+++^*p* < 0.0003 vs. *N. luridiformis* MEOH, ^e*ee*^*p* < 0.0003 vs. *N. luridiformis* MEOH).

Figure 3 shows the total phenolic and orthodiphenols compounds in aqueous and methanolic extracts of *B. edulis* and *N. luridiformis*, respectively. The results showed that there were significant differences between aqueous (24.74 ± 1.11 mg GAE.g^−1^ DW) and methanolic (21.62 ± 0.77 mg GAE.g^−1^ DW) extracts of *B. edulis*. Similar results were observed between aqueous (7.64 ± 0.08 mg GAE.g^−1^ DW) and methanolic (5.64 ± 0.56 mg GAE.g^−1^ DW) extracts of *N. luridiformis*. Noteworthy, the amount of total phenolic compounds content in the aqueous *B. edulis* extract was significantly higher than in the aqueous extract of *N. luridiformis*. Like the total phenolic compounds, the orthodiphenols content in the aqueous extract of *B. edulis* (30.54 ± 4.23 mg GAE.g^−1^ DW) was significantly higher than in the aqueous extract of *N. luridiformis* (13.50 ± 1.90 mg GAE.g^−1^ DW). On the other hand, no significant differences (*p* > 0.05) were observed between the aqueous and methanolic extracts of *B. edulis*.

#### In-vitro Antioxidant Activity

**Figure 4:**
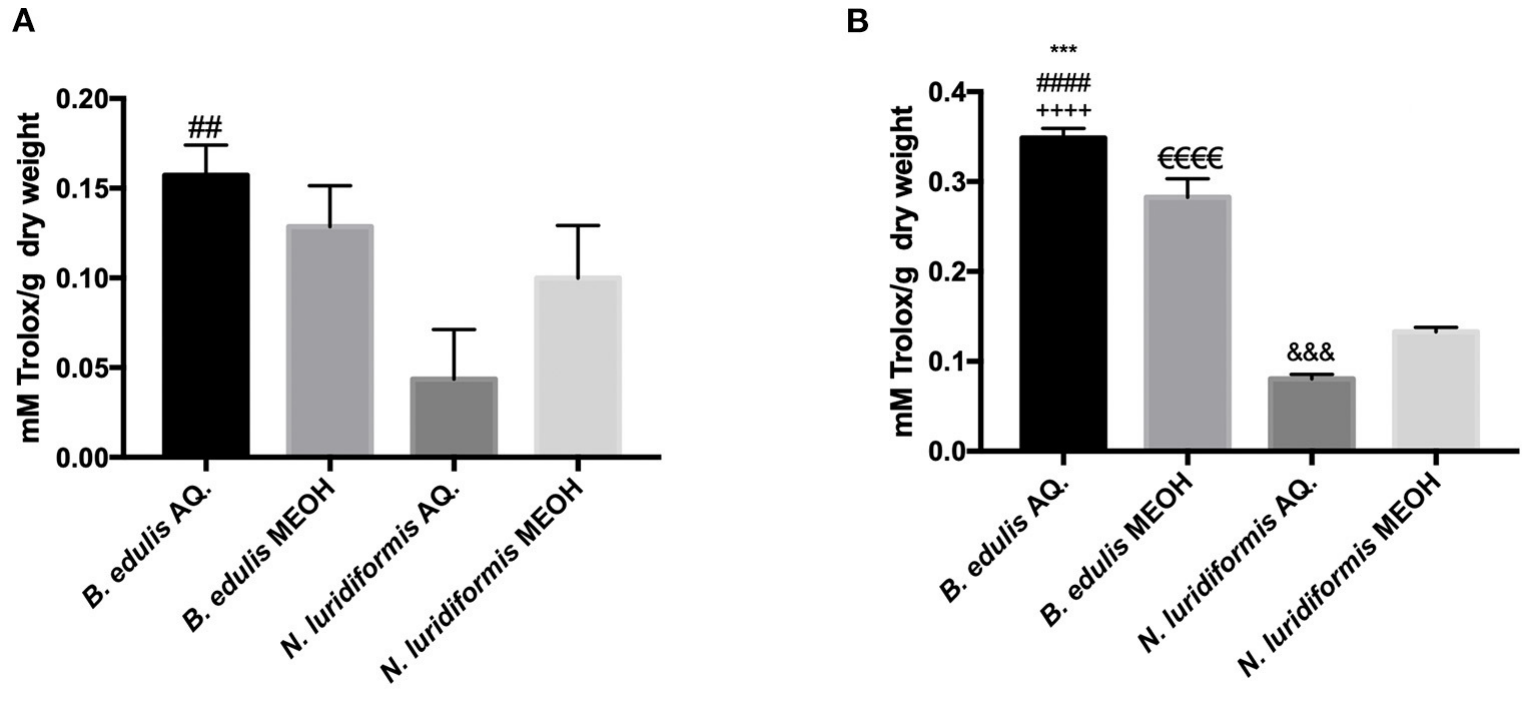
**(A)** 2,2'-azinobis(3-ethylbenzothiazoline-6-sulfonic acid (ABTS) AQ. and MEOH extracts of *B. edulis* and *N. luridiformis* (^##^*p* < 0.0001 vs. *N. luridiformis* AQ.); **(B)** Ferric reducing antioxidant power (FRAP) AQ. and MEOH extracts of *B. edulis* and *N. luridiformis* (***p < 0.0003 vs. *B. edulis* MEOH, ^####^p <0.0001 vs. *N. luridifomis* AQ., ^++++^*p* < 0.0001 vs. *N. luridiformi*s MEOH, ^e*eee*^*p* < 0.0001 vs. *N. luridiformi*s MEOH, ^&&&^*p* < 0.0008 vs. *N. luridiformis* MEOH).

**Figure 5:**
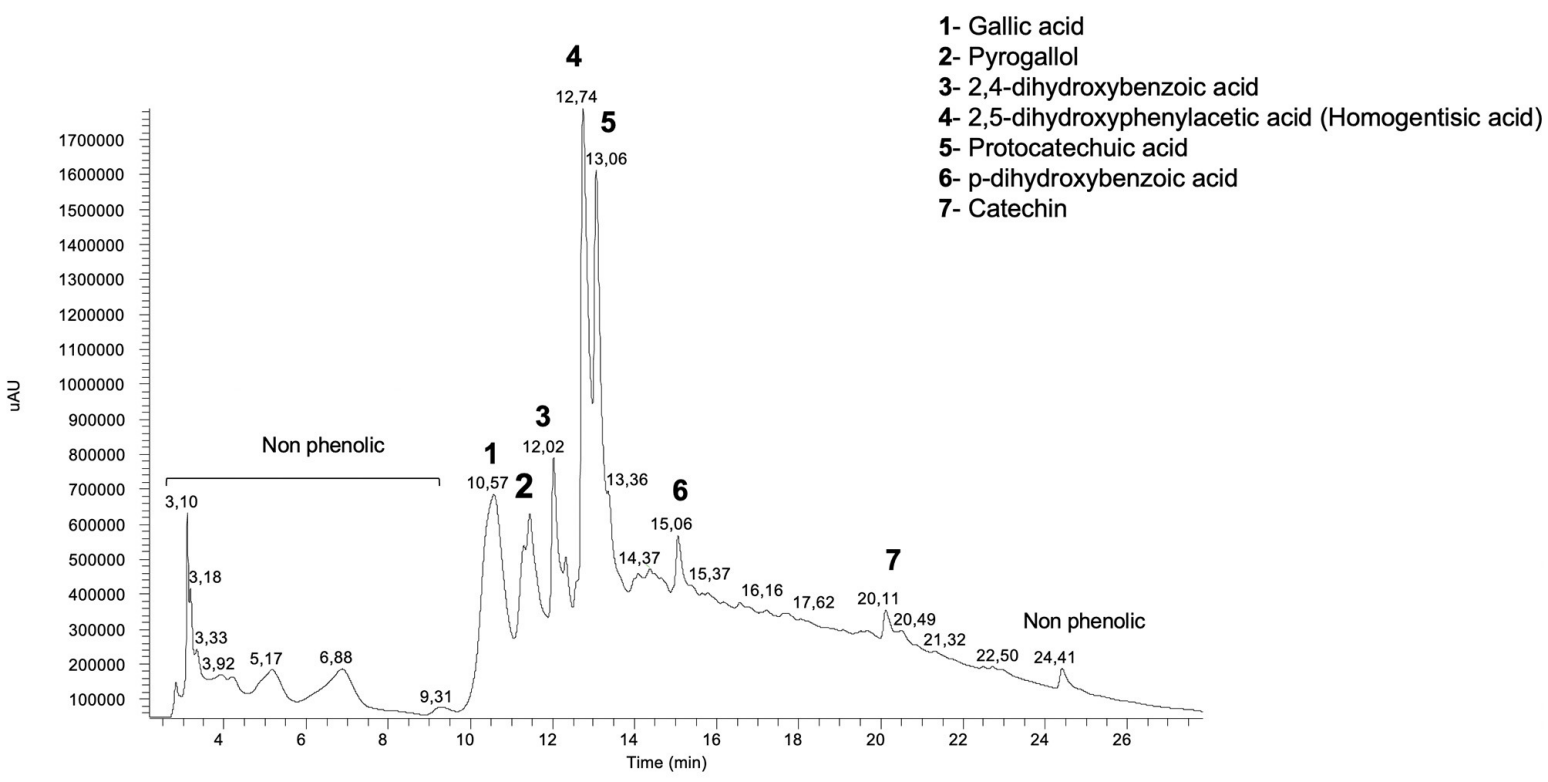
Effect of aqueous and methanolic extracts of B. edulis and N. luridiformis on viability of human foreskin fibroblasts-1 (HFF-1). Cell viability following incubation with indicated concentrations of crude extract for 24 h was determined using the MTT assay. Cell viability is expressed as a percentage of untreated cells. Results shown in the graphs are mean ± SD obtained from triplicate experiments.

According to the previous reports, mushrooms polyphenols exhibit a variety of bioactivities, especially antioxidant properties (6). Consequently, with different approaches and mechanisms, the 2 main usual antioxidant/antiradical activity assays, FRAP and ABTS, were carried out *in vitro*. The results obtained are shown in Figure 5. The aqueous extracts from both the species showed a higher antioxidant activity compared to the methanolic extracts. Also, a higher ABTS^•+^ radical scavenging activity was observed for the aqueous extract of *B. edulis* (0.157 ± 0.02 mmol Trolox.g^−1^ DW) when compared with the methanolic extract (0.128 ± 0.02 mmol Trolox.g^−1^ DW). Noteworthy, a significant difference (*p* > 0.05) between the aqueous extract of *B. edulis* and the aqueous extract of N. luridiformis (0.044 ± 0.02 mmol Trolox.g^−1^ DW) was observed. The reducing power of ferrous ions in the tested extracts presents similar results to the radical scavenging capacity (Figure 4). The FRAP capacities of the 4 extracts were in the order of *B. edulis* aqueous (0.349 ± 0.01 mmol Trolox.g^−1^ DW) > *B. edulis* methanolic (0.282 ± 0.02 mmol Trolox.g^−1^ DW) > *N. luridiformis* methanolic (0.133 ± 0.005 mmol Trolox.g^−1^ DW) > *N. luridiformis* aqueous (0.08 ± 0.005 mmol Trolox.g^−1^ DW). These results are in accordance with those obtained for the total phenolic compounds and orthodiphenols contents of methanolic and aqueous extracts.

#### Polyphenol Composition

In this study, water and methanol extracts were used to assess polyphenol composition of the species of mushroom. The results obtained by HPLC-DAD analysis of the mushrooms extracts are given in Table 5 and *B. edulis* aqueous chromatogram can be seen in Figure 5. Differences in both the polyphenol profile and content between water and methanol extracts were found. A total of nine different polyphenols were identified. Aqueous extract of *B. edulis* profile exhibited high content of protocatechuic acid (18.1 ± 1.8 mg 100 g^−1^ DW) and homogentisic acid (14.4 ± 2.7 mg 100 g^−1^ DW). Other polyphenols were gallic acid, pyrogallol, 2,4-dihydroxybenzoic acid, p-dihydroxybenzoic acid, and catechin. Protocatechuic acid (11.5 ± 3.2 mg 100 g^−1^ DW) and homogentisic acid (6.2 ± 2.2 mg 100 g^−1^ DW) were also the main polyphenols found in methanolic extracts of *B. edulis*, followed by gallic acid, p-dihydroxybenzoic acid, and catechin. Protocatechuic acid (19.6 ± 3.6 mg 100 g^−1^ DW) was the main polyphenol found in the aqueous extract of *N. luridiformis*, followed by the homogentisic acid and 2,4-dihydroxybenzoic acid. The 2,4-dihydroxybenzoic acid (7.0 mg ± 1.5 100 g^−1^ DW) was the main polyphenol identified in the methanolic extract of *N. luridiformis* followed by protocatechuic acid, homogentisic acid, 4-hydroxybenzoic acid, and vanillic acid. In general, *B. edulis* showed higher content of individual polyphenols compared to other species, which corroborates the findings of total phenolics and orthodiphenols contents. In addition, our findings, showed for all the samples the presence of important polyphenols such as protocatechuic acid, homogentisic acid, gallic acid, and 2,4-dihydroxybenzoic acid and others, often reported by literature as having important antioxidant and antimicrobial activities.

**Table 5:** Phenolic compounds identified and quantified in the aqueous and methanolic extracts of *B. edulis* and *N. luridiformis*.

**Mushrooms extracts**	**Phenolic compounds**	**[.] mg. g^**−1**^ dry weight**
*Boletus edulis* aqueous extract	Gallic acid	7.3 ± 1.2
	Pyrogallol	7.6 ± 0.8
	2,4-dihydroxybenzoic acid	0.5 ± 0.1
	2,5-dihydroxyphenylacetic acid (Homogentisic acid)	14.4 ± 2.7
	Protocatechuic acid	18.1 ± 1.8
	p-dihydroxybenzoic acid	1.8 ± 0.9
	Catechin	2.0 ± 1.4
*Boletus edulis* methanolic extract	Gallic acid	4.3 ± 1.1
	2,5-dihydroxyphenylacetic acid (Homogentisic acid)	6.2 ± 2.2
	Protocatechuic acid	11.5 ± 3.2
	p-dihydroxybenzoic acid	1.3 ± 0.3
	Catechin	0.9 ± 0.1
*Neoboletus luridiformis* aqueous extract	2,4-Dihydroxybenzoic acid	3.8 ± 1.7
	2,5-dihydroxyphenylacetic acid	2.4 ± 1.2
	Protocatechuic acid	19.6 ± 3.6
*Neoboletus luridiformis* methanolic extract	4-Hydroxybenzoic acid	1.9 ± 0.9
	2,4-dihydroxybenzoic acid	7.0 ± 1.5
	2,5-dihydroxyphenylacetic acid (Homogentisic acid)	2.7 ± 0.8
	Protocatechuic acid	5.8 ± 1.3
	Vanillic acid	1.4 ± 0.7

### Cytotoxicity

**Figure 6:**
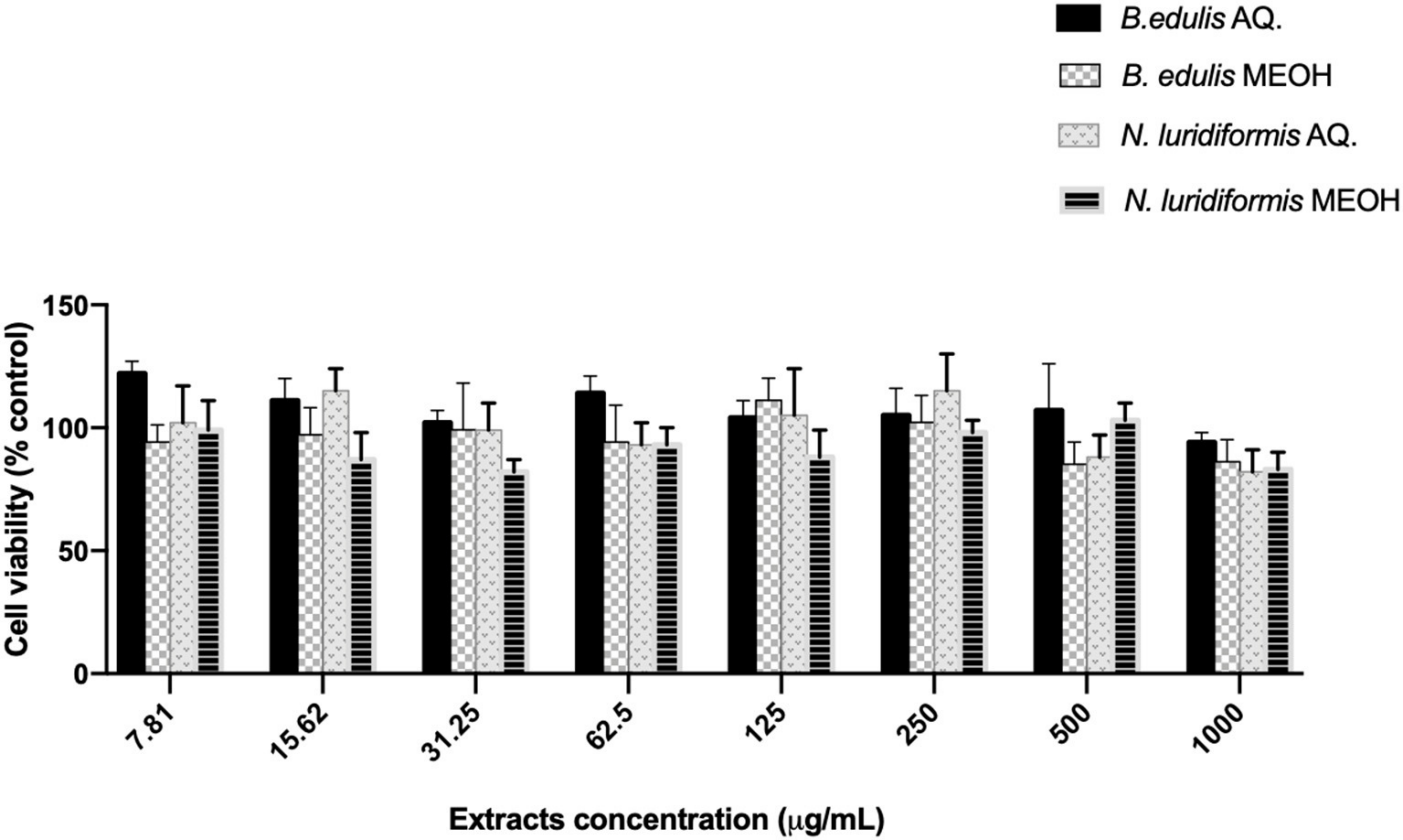
Effect of aqueous and methanolic extracts of *Boletus edulis* and *Neoboletus luridiformis* on viability of Human foreskin fibroblasts (HFF-1). Cell viability following incubation with indicated concentrations of crude extract for 24 h was determined using the MTT assay. Cell viability is expressed as a percentage of untreated cells. Results shown in the graphs are mean ± SD obtained from triplicate experiments.

Data of Figure 6 indicate that the cell viability of HFF-1 cell lines, after exposure to all extracts in the different concentrations, showed a cell survival rate of ~90%.

## Discussion

Nowadays, the incidence of human pathogens resistant to numerous antibiotics has been increased worldwide. More importantly, the efficacy absence of the conventional antibiotics has generated serious problems on the treatment of infectious diseases (12). Therefore, urge to find new alternative therapies to fighting or decrease cases of infectious diseases was associated with MDR pathogens. Due to the recognized safe status of natural products, the industry interest in antimicrobials derived from nature has boosted (13). Indeed, an outstanding amount of antimicrobial has been obtained from natural sources including mushrooms (7). Mushrooms have been used since 1,000 of years in traditional medicine for several diseases and presently continue to play an important role in the discovery of new molecules (14). Mushrooms contain a multiplicity of bioactive compounds with chemical and structural variation that make them versatile in terms of biological activities including antimicrobial action (6). In this sense, this study provides new information with respect to the antimicrobial and antibiofilm activities, chemical composition, and characterization of 2 mushrooms species, *B. edulis* and *N. luridiformis*. To the best of our knowledge, this study provides the first report on the phenolic composition of *N. luridiformis* as well as antimicrobial activity. Moreover, this is a pioneer study with respect to *B. edulis* and *N. luridiformis* antibiofilm properties and constitutes an important step toward to combat the emergent biofilms activities that differ substantially from free-living bacterial cells (9).

The antimicrobial activity evaluation revealed that aqueous and methanolic extracts of both the species of mushrooms were effective against Gram-positive bacteria [methicillin-susceptible Staphylococcus aureus (MSSA) and MRSA] and Gram-negative bacteria (*P. aeruginosa* and *E. coli*). Noteworthy, the aqueous extracts from *B. edulis* presented the highest antimicrobial activity that was obtained against to MRSA MJMC 583 and *P. aeruginosa* carbapenem-resistant MJMC 540, which are in accordance with our previous study (6). Noteworthy, as clinical wound infection EKAPE isolates that are associated to healthcare infections, these results confirmed the overwhelming importance of mushrooms as a source of antimicrobial compounds. Corroborating our findings, a study has found out that water extracts were better than methanol extracts against bacterial and fungal pathogens (15). Similarly, water extracts of wild mushrooms showed to be more effective against *P. aeruginosa, E. coli, B. subtilis*, and *C. albicans* isolates (16), which support our results. It is worth underlining the effect of different solvents on antioxidant activity. In this study, aqueous extracts had markedly higher antioxidant capacities than the methanolic extracts. Therefore, water is a more effective solvent for the extraction of antioxidant compounds from wild mushrooms. Moreover, the antimicrobial differences between the species of mushroom and the two solvents employed (aqueous and methanol) may be due to the contents of phenolic compounds, which was higher for the aqueous extract of *B. edulis*. In fact, several authors have previously associated with the antimicrobial activity of different natural sources to phenolic compounds (17–19).

Concerning to phenolic compounds profile, there are few studies with respect to the individual phenolics in wild edible mushrooms (20). Commercial species are better known in terms of composition; however, wild mushrooms are scarcely studied and, to the best of our knowledge, the polyohenol composition of the species *N. luridiformis* has not been previously described. Data showed that 2,4-dihydroxybenzoic acid, homogentisic acid, and protocatechuic acid were the only three polyphenols common to both the aqueous and methanolic extracts. Protocatechuic acid was present in higher amounts in the aqueous extract, while high levels of 2,4-dihydroxybenzoic acid were detected in the methanolic extract. 4-hydroxybenzoic acid and vanillic acid were only present in methanolic extracts. For *B. edulis*, the experimental data are comparable to those reported in previous studies (20, 21); however, specific and characteristic composition of each mushroom may be associated with several factors, namely, genetic, physiological, and morphological characteristics, agroclimatic conditions, and ripening stage (6). The major phenolic compounds in aqueous extract were protocatechuic acid and homogentisic acid. Besides protocatechuic acid and homogentisic acid, the aqueous extract also exhibited considerable levels of gallic acid, pyrogallol, and catechin. On the other hand, the methanolic extract showed similar phenolic composition, however, in low contents, except for 2,4-dihydroxybenzoic acid and pyrogallol, which are only present in aqueous extracts. Noteworthy, the proportion of protocatechuic acid, however, is higher than in previous studies from different geographical origins (20, 22).

Alves et al. (4) confirmed that phenolics were the most important active compounds against bacteria (23). They also identified 2,4-dihydroxybenzoic and protocatechuic acids as the main phenolic compounds with higher activity against the majority of Gram-negative and Gram-positive bacteria (23). Moreover, this study highlighted the importance of the carboxylic group in the molecular structure for antimicrobial activity. In line with this, this study showed that the aqueous extract of *B. edulis* presented protocatechuic acids as the main compound, followed by homogentisic acid, pyrogallol, and gallic acid. It should be noted that protocatechuic acid as well as homogentisic acid has carboxylic groups, which may contribute for the higher antimicrobial activity. These compounds were also present in the other extracts tested, however, in low contents, explaining the highest aqueous *B. edulis* antimicrobial activity. In addition, gallic acid has been identified as the active compound for the inhibition of *E. coli, Salmonella enteritidis* (24), *P. aeruginosa, S. aureus*, and *Listeria monocytogenes* (25). Structural activity of correlation assays revealed that the three hydroxyl groups of gallic acid were effective for antibacterial activity and all the substituents of the benzene rings were effective against *S. aureus* (24). Gallic acid was only identified in *B. edulis*; however, the aqueous extracts showed an amount almost twice higher than the methanolic extract, which may explain the better antimicrobial activity of the aqueous extract.

The different phytochemical composition as well as antioxidant activity caused different effectiveness in the Gram-negative and Gram-positive biofilms. The percentages of biomass removal and inactivation were always higher for *E. coli* than for *S. aureus* for all the extracts tested. The total biofilm removal was not achieved with any of the extracts; however, the highest reduction (94%) in biomass removal was found for *E. coli* with the aqueous extract of *B. edulis*. Greater effect of extracts on biofilms of *E. coli* than on *S. aureus* is due to morphology, which is known to be different; generally, *S. aureus* biofilms are denser than those of *E. coli* (26). To the best of our knowledge, the antibiofilm properties of *B. edulis* and *N. luridiformis* have not been reported before and, therefore, our results can be evaluated as the first report about properties of these unique and wild species. Furthermore, and more importantly, all the extracts analyzed had no cytotoxic effect on the HFF-1 cell lines, which demonstrates their potential in the management and treatment of wound.

It should be point out that specific compounds must be evaluated for further conclusions.

Finally, the antimicrobial and antibiofilm activities obtained in *Boletus* spp. extracts, especially against ESKAPE pathogens, is a capable finding to fight the most frequent causes of bacterial wound infections in developed countries. This study also provides comparative data on the potential bioactive compounds of different wild mushrooms and different solvents to help select the best system for antimicrobial application.

## Data Availability Statement

The raw data supporting the conclusions of this article will be made available by the authors, without undue reservation.

## Author Contributions

MJS, GM, and JG proposed the subject and designed. JG and FC carried out the chemical and the antimicrobial experiments. FR carried out the study of cytotoxicity. AA carried out the chemical composition of the extracts. GM provided fungal samples and funding acquisition. JG, FR, and MJS wrote the manuscript. All the authors reviewed and contributed to the manuscript.

## Funding

This study was funded by the project I&T Companies in Co-promotion FungiTech, Norte-01-0247-FEDER-033788; National Funds by FCT—Portuguese Foundation for Science and Technology, under the project UIDB/04033/2020 (CITAB-Center for the Research and Technology of Agro-Environmental and Biological Sciences).

## Conflict of Interest

The authors declare that the research was conducted in the absence of any commercial or financial relationships that could be construed as a potential conflict of interest.

## Publisher's Note

All claims expressed in this article are solely those of the authors and do not necessarily represent those of their affiliated organizations, or those of the publisher, the editors and the reviewers. Any product that may be evaluated in this article, or claim that may be made by its manufacturer, is not guaranteed or endorsed by the publisher.
